# Clinical and Biological Correlates of Resistance and Relapse After CD19-Directed CAR T-Cell Therapy in Large B-Cell Lymphoma: A Structured Narrative Review

**DOI:** 10.3390/cancers18203370

**Published:** 2026-10-18

**Authors:** Jean Silva de Souza Resende, Igor Samesima Giner, Maria Gabriela Custodio de Figueiredo, Mariana Martins Paraizo, Enzo Lalli, Bonald Cavalcante de Figueiredo

**Affiliations:** 1 Instituto de Pesquisa Pelé Pequeno Príncipe, Curitiba 80250-060, PR, Brazil; jean.resende@professor.fpp.edu.br (J.S.d.S.R.); igor.giner@professor.fpp.edu.br (I.S.G.); gabi.figueiredo@mail.utoronto.ca (M.G.C.d.F.); mariana.paraizo@pelepequenoprincipe.org.br (M.M.P.); 2 Faculdades Pequeno Príncipe, Rebouças, Curitiba 80230-020, PR, Brazil; 3 Bioinformatics and Systems Biology Laboratory, Federal University of Paraná, Curitiba 81520-260, PR, Brazil; 4 Institut de Pharmacologie Moléculaire et Cellulaire CNRS UMR7275, Inserm U1423, Université Côte d’Azur, 06560 Valbonne, France; lalli@ipmc.cnrs.fr

**Keywords:** antigen escape, T-cell exhaustion, cellular fitness, immune evasion, tumor microenvironment, predictive biomarkers, spatial transcriptomics

## Abstract

Background/Objectives: CD19-directed chimeric antigen receptor (CAR) T-cell therapy has transformed the treatment of relapsed/refractory large B-cell lymphoma (r/r LBCL), yet primary resistance and relapse remain common. We evaluated how recent clinical and translational evidence refines established resistance frameworks and which knowledge gaps prevent the relative contribution of individual mechanisms from being determined. Methods: We conducted a structured narrative review of 14 selected clinical and translational studies addressing tumor-, product-, host-, and microenvironment-related dimensions of treatment failure, synthesized across four interpretive domains and compared with nine previous reviews. Results: Durable benefit was associated with favorable tumor and immune transcriptional programs and greater CAR T-cell fitness, expansion, or persistence. Treatment failure was associated with tumor-intrinsic resistance programs, exhausted T-cell states, systemic inflammatory signals, and suppressive tumor-microenvironment features, including PD-L1-expressing macrophages. Antigen escape remained relevant, but incomplete serial CD19 assessment precluded conclusions about its relative contribution. Comparison with earlier reviews showed continuity rather than replacement, with LBCL-focused evidence adding biological resolution. Conclusions: Resistance and relapse after CD19 CAR T-cell therapy in LBCL are multifactorial. Prospective longitudinal studies integrating tumor tissue, apheresis and manufactured products, peripheral blood, and matched relapse samples are needed to validate predictive biomarkers and guide rational treatment strategies.

## Simple Summary

CD19-directed chimeric antigen receptor (CAR) T-cell therapy has transformed the treatment of large B-cell lymphoma (LBCL), but many patients either do not respond or subsequently relapse. This structured narrative review examines clinical outcomes and biological factors associated with resistance and relapse. Across 14 studies, treatment failure could not be attributed to loss of CD19 alone, although incomplete assessment of CD19 at relapse limits this interpretation. Tumor-intrinsic programs, impaired CAR T-cell fitness and persistence, systemic inflammation, and an immunosuppressive tumor microenvironment also contributed, although differences in study design and incomplete testing at relapse prevented determination of the relative importance of each mechanism. Comparison with previous reviews shows continuity with earlier frameworks, with greater biological resolution in LBCL rather than replacement of the antigen-escape model. These findings support longitudinal assessment of the tumor, infused cell product, and peripheral immune response, and integrated biomarkers to identify high-risk patients and guide rational treatment strategies.

## 1. Introduction

Diffuse large B-cell lymphoma (DLBCL), the most common subtype of large B-cell lymphoma (LBCL), historically had limited therapeutic options once patients developed relapsed or refractory (r/r) disease. The development of CD19-directed chimeric antigen receptor (CAR) T-cell therapy represented a major advance and transformed the therapeutic landscape of r/r LBCL [1,2].

Advances in adoptive T-cell engineering and CAR design led from first-generation constructs to clinically effective second-generation CAR T cells containing costimulatory domains that enhance activation, proliferation, and persistence [3]. This work culminated in the regulatory approval of three second-generation CD19 CAR T-cell products for LBCL: axicabtagene ciloleucel (axi-cel) [4], tisagenlecleucel (tisa-cel) [5], and lisocabtagene maraleucel (liso-cel) [6].

These products differ in their costimulatory signaling: axi-cel uses a CD28 domain, whereas tisa-cel and liso-cel use 4-1BB, a distinction that may influence expansion kinetics and persistence. Other approaches include regionally approved CD19-directed products, such as inaticabtagene autoleucel [7], as well as investigational allogeneic products and engineering strategies designed to improve activity and durability through dual targeting, additional costimulatory signals, or cytokine-secreting armored CAR T cells [8].

Failure after CAR T-cell therapy is multifactorial and may arise from tumor-intrinsic, host-related, treatment-related, and CAR T-cell-associated factors. Antigen escape is one recognized pathway: tumor cells may evade CAR-mediated recognition through reduced or absent CD19 expression, acquired CD19 alterations, aberrant splicing, selection of pre-existing antigen-negative subclones, lineage switching, epitope masking, or trogocytosis [9]. Although these mechanisms are biologically important, their frequency and relative contribution in LBCL remain difficult to establish because CD19 expression is not assessed uniformly or longitudinally across studies.

Relapse with preserved CD19 expression points to additional mechanisms. Characteristics of the starting T-cell compartment and manufactured product, in vivo CAR T-cell expansion and persistence, and the development of dysfunctional or exhausted states may all affect response durability. At the same time, tumor-intrinsic transcriptional programs, systemic inflammation, and an immunosuppressive tumor microenvironment can limit CAR T-cell trafficking and function. Inhibitory pathways involving PD-1/PD-L1, LAG-3, and TIM-3, together with suppressive myeloid-cell populations, have therefore emerged as candidate correlates of nonresponse or relapse [10,11]. However, associations reported in small and heterogeneous cohorts should not be interpreted as establishing a single dominant mechanism.

For the purposes of this review, resistance is used as an overarching biological concept encompassing both primary resistance and mechanisms that enable subsequent disease recurrence. Primary resistance, or refractory disease after CAR T-cell infusion, refers to failure to achieve an objective response or to the presence of persistent or progressive disease at the first formal response assessment. Relapse refers to disease recurrence or progression after an initial response, particularly after complete remission. These outcomes may arise through overlapping mechanisms involving tumor-intrinsic characteristics, loss or alteration of CD19 expression, impaired CAR T-cell fitness, insufficient expansion or persistence, systemic inflammatory factors, and an immunosuppressive tumor microenvironment. Because definitions, response-assessment criteria, and evaluation time points varied among the included studies, the terminology and classifications used by the original investigators were retained when individual study findings were summarized.

Although previous reviews have established that failure after CD19-directed CAR T-cell therapy can involve antigen escape, inadequate CAR T-cell fitness or persistence, host-related factors, and an immunosuppressive tumor microenvironment, important gaps remain in the LBCL-specific evidence. Several earlier syntheses included heterogeneous B-cell malignancies, emphasized B-cell acute lymphoblastic leukemia or CD19-negative relapse, or preceded recent studies using transcriptomic, spatial, metabolic, and cytokine-based analyses. Moreover, the extent to which these biological findings are linked to clinical outcomes, arise from distinct biological compartments, or derive from overlapping trial populations has not been consistently examined. We therefore conducted a structured narrative review of original clinical and translational studies in r/r LBCL to relate study-level outcomes to biological correlates measured in pretreatment tumor tissue, apheresis material, manufactured CAR T-cell products, peripheral blood, and relapse samples. We also compared these findings with previous reviews to determine how recent LBCL evidence refines established resistance frameworks and to identify the methodological gaps that currently prevent the relative contribution of individual mechanisms from being determined.

## 2. Materials and Methods

### 2.1. Study Design

This study was conducted as a structured narrative review. Its purpose was not to identify exhaustively every publication concerning outcomes after CD19-directed CAR T-cell therapy. Instead, it aimed to integrate a selected group of original clinical and translational studies addressing complementary biological levels of resistance or relapse in adults with relapsed or refractory LBCL and to compare the resulting synthesis with the conceptual frameworks of previous reviews.

The original studies were examined across four interrelated levels: tumor-intrinsic characteristics and antigen expression; the composition and functional state of the apheresis material and manufactured CAR T-cell product; in vivo CAR T-cell expansion, persistence, and dysfunction; and host-related or tumor-microenvironmental determinants of treatment failure. Clinical and interventional studies were also considered when they established the relevant outcome context or evaluated strategies directed at a proposed resistance pathway.

### 2.2. Literature Identification and Study Selection

Relevant publications were identified through iterative searches of PubMed/MEDLINE, Embase, Web of Science, and Scopus, supplemented by examination of reference lists and citation tracking (Supplementary Table S1). Search concepts included CD19-directed CAR T-cell therapy, large B-cell lymphoma, diffuse large B-cell lymphoma, resistance, primary nonresponse, relapse, antigen escape, T-cell fitness, exhaustion, expansion, persistence, transcriptomic signatures, cytokines, and the tumor microenvironment.

Original studies were selected purposively according to their relevance to the biological framework of the review. Priority was given to peer-reviewed studies in adults with LBCL that related clinical response, resistance, relapse, progression, or response durability to biological findings obtained from pretreatment tumor tissue, apheresis material, the manufactured CAR T-cell product, peripheral blood, or relapse samples. Studies evaluating interventions intended to overcome a proposed resistance pathway or providing essential clinical context were also considered.

The selected studies were intended to provide complementary biological and clinical perspectives rather than an exhaustive catalogue of all studies meeting formal eligibility criteria. Consequently, the review does not provide pooled estimates, a PRISMA study-selection flow diagram, or a formal risk-of-bias assessment.

### 2.3. Information Extraction and Narrative Synthesis

For each selected study, we extracted the study design, patient population, CAR T-cell product or intervention, relevant analytical cohort, clinical outcomes, biological material and analytical platform, principal biological findings, and proposed relationship with treatment response or failure. Outcomes from parent clinical trials were distinguished from those of selected translational or biomarker subsets.

The extracted variables included author and year of publication; study design and setting; characteristics and size of the relevant analytical cohort; patient demographics and LBCL subtypes; CAR T-cell product; treatment setting; criteria used to define response, resistance, or relapse; overall response rate (ORR); complete response (CR) and partial response (PR) rates; overall survival (OS); progression-free survival (PFS); event-free survival (EFS), when reported; duration of response (DOR); relapse frequency; CD19 expression at relapse; CAR T-cell expansion and persistence; and the biological markers or molecular signatures associated with treatment outcome.

To account for biological and methodological heterogeneity, biomarker data were categorized according to their source and analytical platform. These categories included pretreatment tumor tissue, the apheresis product, the manufactured CAR T-cell product, peripheral blood, and relapse samples, as well as flow cytometry, immunohistochemistry or immunofluorescence, bulk RNA sequencing, single-cell RNA sequencing, spatial transcriptomics, and other molecular assays. Outcomes from parent trials were distinguished from those reported for selected translational or biomarker cohorts to prevent the attribution of parent-study efficacy estimates to nonrepresentative analytical subsets.

The findings were synthesized narratively across the four biological levels defined above. Nine previous reviews were then compared according to disease focus, classification of treatment failure, mechanisms emphasized, integration of clinical and molecular evidence, and translational contribution. This comparison was used to determine how recent LBCL-focused evidence adds biological resolution to, or qualifies, previously proposed frameworks.

### 2.4. Methodological Appraisal

Because this was a structured narrative rather than a systematic review, no formal risk-of-bias instrument was applied. Instead, methodological features expected to influence the reliability of reported associations (study design, sample size, cohort selection, retrospective versus prospective design, and exploratory versus prespecified analyses) were appraised qualitatively by the reviewers and considered when interpreting the findings.

### 2.5. Data Synthesis

The findings of the included studies are presented in thematic groups within Section 3. However, to synthesize these data, our Discussion is organized across four interrelated biological levels that serve as an interpretive framework: (1) tumor-intrinsic characteristics and antigen escape; (2) characteristics of the starting T-cell compartment and manufactured CAR T-cell product; (3) in vivo CAR T-cell expansion, persistence, and functional state; and (4) host-related and tumor-microenvironmental determinants of treatment failure. Clinical outcomes were interpreted within the relevant analytical cohort, and results from selected biomarker subsets were distinguished from outcomes of their respective parent trials. The selected publications were classified according to their role in the narrative synthesis. Mechanistic or translational studies directly examined associations between biological characteristics and treatment response, resistance, relapse, or response durability. The evaluated characteristics originated from pretreatment tumor tissue, the tumor microenvironment, apheresis material, the manufactured CAR T-cell product, peripheral blood, or post-treatment samples. Clinical-context studies established the relevant efficacy and failure setting, evaluated clinical modifiers such as age or lymphodepletion, provided parent-trial outcomes for selected biomarker cohorts, or tested interventions directed at proposed resistance pathways. Clinical-context studies were not considered direct evidence of a resistance mechanism unless they included an explicit biological analysis supporting such an interpretation.

Finally, the findings from the 14 original studies were compared narratively with the objectives, principal contributions, and limitations of nine previous reviews. This contextual comparison was used to assess how the LBCL-focused evidence supports, modifies, or extends earlier models of relapse centered predominantly on CD19 antigen escape.

## 3. Results

### 3.1. Overview and Characteristics of the Included Original Studies

Fourteen original studies were selected for their relevance to the clinical and multilevel biological framework of the review. The evidence base comprised prospective clinical trials, translational and correlative analyses, and retrospective real-world cohorts that evaluated CD19-directed CAR T-cell therapy or related immunomodulatory strategies in patients with relapsed or refractory B-cell malignancies. The included studies differed substantially in the treatment-failure phenotype evaluated. Some investigated primary nonresponse using response status at an early or predefined assessment, whereas others examined progression or relapse after an initial response. Several studies used composite or time-to-event outcomes that combined patients with primary refractory disease and those who relapsed after achieving a response. Therefore, findings were classified, whenever possible, as relating to primary nonresponse, post-response relapse, or a mixed or undifferentiated treatment-failure endpoint. Associations derived from the latter category could not be assigned specifically to either primary resistance or relapse.

Most studies evaluated patients with DLBCL, although several cohorts also included high-grade B-cell lymphoma, transformed follicular lymphoma, other LBCL subtypes, or follicular lymphoma. Clinical cohorts ranged from 12 to 359 patients, whereas biomarker studies frequently analyzed selected patient subsets or tissue collections. Age reporting was inconsistent, and Ram et al. specifically evaluated patients aged 70 years or older [12]. Locke et al. randomized 359 patients in ZUMA-7 (180 to axi-cel and 179 to standard of care [SOC]) [13]. The subsequent analysis by Tian et al. included pretreatment gene-expression data from 134 axi-cel-treated patients [14]. Several publications reanalyzed overlapping ZUMA-1 or ZUMA-7 populations; consequently, their sample sizes cannot be summed to obtain a unique-patient total.

The evaluated interventions included commercial CD19 CAR T-cell products, CAR T cells combined with checkpoint inhibition, checkpoint blockade administered after CAR T-cell failure, and an alternative lymphodepletion regimen. Other studies focused on pretreatment tumor biology, the fitness of the apheresis T-cell starting material, circulating cytokines, or the spatial organization of the tumor microenvironment. The principal characteristics of all 14 studies are summarized in Table 1.

Clinical outcomes were reported heterogeneously because the included publications differed substantially in design, population, intervention, analytic purpose, and follow-up. CR, PR, ORR, relapse or progression, OS, PFS, event-free survival (EFS), and DOR were therefore synthesized descriptively rather than pooled (Table 2). The findings are presented below in thematic groups that integrate clinical outcomes with the biological questions addressed.

### 3.2. Clinical Efficacy and Treatment-Context Studies

Locke et al. reported the landmark phase III ZUMA-7 trial, in which second-line axi-cel was superior to SOC salvage chemoimmunotherapy followed, in responding patients, by high-dose therapy and autologous stem-cell transplantation in patients with relapsed or refractory large B-cell lymphoma [13]. Axi-cel produced a CR rate of 65% and an ORR of 83%, with a median EFS of 8.3 months versus 2.0 months with SOC. The estimated 24-month EFS rates were 41% and 16%, respectively. Nevertheless, progression remained frequent, showing that improved population-level efficacy did not eliminate primary resistance or early relapse.

Ram et al. compared 41 patients aged at least 70 years with 41 matched younger patients treated with axi-cel or tisagenlecleucel [12]. In the older group, the CR rate was 46%, the ORR was 63%, and 12-month OS was 69%; outcomes and CAR T-cell expansion were broadly comparable between age groups. Baseline T-cell phenotype, including CD4/CD8 distribution and markers of differentiation or exhaustion, also did not show a major age-related disadvantage. High lactate dehydrogenase and rapidly progressive disease were more informative adverse features, suggesting that disease kinetics rather than chronological age may limit efficacy.

Ghilardi et al. retrospectively compared lymphodepletion with bendamustine (*n* = 90) versus fludarabine/cyclophosphamide (Flu/Cy; *n* = 42) before tisagenlecleucel in 132 patients with relapsed or refractory LBCL treated consecutively at three centers [18]. The best ORR was similar with bendamustine and Flu/Cy (50.0% versus 42.9%; *p* = 0.444), as was median PFS (3.26 versus 3.06 months; *p* = 0.210), at a median follow-up of 24.7 months. Median OS was not reached in the bendamustine group and was 10.23 months in the Flu/Cy group (*p* = 0.030), although this nonrandomized comparison may have been affected by institutional practice and unmeasured confounding. Bendamustine was associated with less profound early lymphocytopenia, lower rates of cytokine release syndrome, neurotoxicity, infections and hematologic toxicity, and reduced hospital utilization. These findings support bendamustine as a feasible alternative lymphodepletion regimen, but randomized studies are required to confirm comparative efficacy and safety.

Abramson et al. reported the 2-year follow-up of TRANSCEND NHL 001, a multicenter, multicohort, open-label, seamless-design Phase 1/2 study evaluating lisocabtagene maraleucel (liso-cel) in 270 heavily pretreated patients with relapsed or refractory large B-cell lymphoma (R/R LBCL) [19]. Liso-cel demonstrated durable clinical activity, yielding an objective response rate of 73% and a complete response rate of 53%, with a median overall survival of 27.3 months in the overall cohort and 48.5 months among complete responders. Severe cytokine release syndrome and neurological events were notably low (2% and 10%, respectively), which was contextually attributed to the precise 1:1 dosing ratio of CD4+ and CD8+ CAR+ T cells designed to minimize product composition variance. However, similar to other practice-changing CAR T-cell trials, the incremental benefit and long-term efficacy remain difficult to isolate definitively due to the lack of a concurrent comparator arm, and the true duration of response and progression-free survival (median 6.8 months) were likely underestimated due to extensive censoring at the 24-month study completion mark.

### 3.3. Tumor-Intrinsic and Tumor-Microenvironment Determinants

Scholler et al. examined pretreatment and post-treatment tumor immune contexture in 51 axi-cel-treated patients from ZUMA-1 [11]. In the parent ZUMA-1 trial, the reported CR and ORR rates were 58% and 83%, respectively, and approximately 39% of patients maintained a response at a median follow-up of 27.1 months. Durable benefit was associated with a pretreatment immune-inflamed microenvironment and higher T-cell density. At relapse, the authors observed increased expression of inhibitory and immunosuppressive markers, together with evidence of antigen-evasion pathways, supporting the contribution of both T-cell dysfunction and tumor adaptation.

Tian et al. performed a post hoc analysis of pretreatment tumor gene-expression data from 134 axi-cel-treated patients in ZUMA-7 [14]. Higher scores for a six-gene expression signature (6-GES) were associated with more favorable EFS (HR = 0.27); PFS (HR = 0.27); DOR (HR = 0.29), whereas higher scores for a 17-gene signature (17-GES) were associated with poorer outcomes, EFS (HR = 6.12); PFS (HR = 7.47); DOR (HR = 7.59).

Corresponding associations were not observed in the SOC group, suggesting potential treatment-specific relevance rather than establishing a validated predictive biomarker. The authors related these signatures to inflammatory signaling, immune evasion, apoptosis regulation, and metabolic conditions; these biological interpretations remained correlative.

Hirayama et al. analyzed pretreatment biopsies from 24 patients receiving JCAR014 or JCAR021, including a small subgroup treated with durvalumab [20]. Nine patients (38%) achieved CR, and most complete responses were durable during the reported observation period. Treatment failure was associated not simply with the abundance of immune cells, but with their spatial organization: close proximity of PD-1-positive T cells to PD-L1-positive macrophages or tumor cells and an intercalated, dysfunctional immune architecture were unfavorable. These findings emphasize localized inhibitory signaling within the tumor microenvironment.

Li et al. integrated single-nucleus RNA and chromatin-accessibility profiling of 232 lymphoma and control biopsies with validation in multiple cohorts, including ZUMA-1 and ZUMA-7 [21]. They defined three large B-cell lymphoma microenvironment archetypes: lymphoid-enriched (LN), fibroblast/macrophage-enriched (FMAC), and T-cell-exhausted (TEX). In ZUMA-7, 12-month EFS after axi-cel was 71% for LN, 41% for FMAC, and 39% for TEX, and the TEX group showed the highest rate of primary progression. These retrospective analyses associated microenvironmental ecosystems with CAR T-cell outcomes, without establishing their independent causal contributions.

Plaks et al. analyzed 100 pretreatment and 20 post-relapse tumor biopsies from patients with refractory LBCL treated with axi-cel in ZUMA-1, including 18 paired samples [16]. Approximately 30% of evaluable relapses were CD19-low or CD19-negative, with 5/18 paired tumors showing substantially reduced CD19 protein at relapse. Conversely, CD19-positive relapse was associated with inadequate CAR T-cell expansion relative to tumor burden.

### 3.4. Premanufacturing T-Cell and Infusion-Product Fitness

Jacobs et al. studied premanufacturing apheresis T cells and liso-cel products from 34 patients with relapsed or refractory large B-cell lymphoma, using a deliberately balanced biomarker cohort of 17 responders and 17 nonresponders [22]. Responders had a higher proportion of CD27-positive CD8-positive memory T cells before manufacturing, whereas the final products were comparatively homogeneous across outcome groups. Higher mitochondrial membrane potential in the starting T cells was associated with more favorable in vivo CAR T-cell expansion. The findings link premanufacturing memory composition and metabolic fitness to subsequent treatment activity, although independent validation is required.

Deng et al. characterized the cellular and transcriptional composition of standard-of-care axi-cel infusion products using single-cell RNA sequencing and capture-based identification of CAR-positive cells. The discovery cohort included 24 patients with r/r LBCL: 16 with DLBCL, six with transformed follicular lymphoma, and two with primary mediastinal B-cell lymphoma [15]. At the three-month assessment, nine patients achieved a complete response (38%), one achieved a partial response (4%), 13 had progressive disease (50%), and one was not evaluable because of death from sepsis. Infusion products administered to complete responders contained approximately threefold higher frequencies of CD8-positive T cells expressing memory-associated signatures, whereas exhausted CD8-positive and CD4-positive T-cell states were enriched in patients with partial response or progressive disease. Early molecular response, measured by circulating tumor DNA seven days after infusion, was significantly associated with subsequent clinical response (*p* = 0.008), and a CD8 T-cell exhaustion signature was strongly associated with an unfavorable molecular response (q = 2.8 × 10^−149^). The study also identified a rare infusion-associated cell population with monocyte-like transcriptional features that was associated with high-grade ICANS and was evaluated in an independent 16-patient validation cohort.

### 3.5. Systemic Inflammatory and Cytokine Determinants

Pu et al. used a multiplex platform to profile 14 circulating cytokines and inflammatory markers in 136 longitudinal plasma samples from 32 patients with DLBCL treated with tisagenlecleucel [24]. The study focused on cytokine release syndrome and EFS rather than conventional tumor-response endpoints. Distinct inflammatory profiles included changes in interleukin-6, C-reactive protein, and transforming growth factor beta 1 (TGF-β1). Elevated baseline TGF-β1 was associated with high-grade cytokine release syndrome and inferior EFS. The associations were univariate, and thresholds were derived within the same small cohort; the findings therefore identify a candidate biomarker rather than establish a causal mechanism of treatment failure.

### 3.6. Checkpoint-Directed Interventions

Chong et al. evaluated pembrolizumab administered after progression following CD19 CAR T-cell therapy in 12 patients [17]. Three patients achieved an objective response, including one CR, and one remission persisted for more than four years; however, most patients progressed. Pembrolizumab induced CAR T-cell re-expansion or activation in a subset, particularly when cells retained activation-associated features and lacked a terminally exhausted phenotype. These findings indicated activity in a subset of patients, but the small, uncontrolled study could not establish which patients would benefit or whether reversal of exhaustion explained the responses.

Jacobson et al. reported ZUMA-6, a single-arm phase I/II study combining axi-cel with the anti-PD-L1 antibody atezolizumab in 28 patients with DLBCL [23]. Durable responses occurred in some patients, but the combination did not show a clear improvement over historical axi-cel experience. Because the study lacked a concurrent axi-cel-only control group, the incremental benefit of atezolizumab could not be established. PD-L2-mediated signaling and alternative inhibitory pathways, including LAG-3 and TIM-3, were proposed as possible explanations for persistent resistance, rather than demonstrated mechanisms of failure.

### 3.7. Integrated Synthesis of Clinical and Biological Evidence

Clinical outcomes and biological associations were synthesized without pooling because the studies differed in CAR T-cell product, treatment setting, analytic population, endpoint definitions, and follow-up. Selected biomarker cohorts were distinguished from their parent trials: in particular, the equal numbers of responders and nonresponders in Jacobs et al. reflected the sampling design and did not represent a treatment response rate [22]. Similarly, the tissue collection analyzed by Li et al. was not a single independent CAR T-cell efficacy cohort [21].

The studies identified complementary associations across several biological levels. Scholler et al., Tian et al., Hirayama et al., and Li et al. related treatment outcomes to tumor immune contexture, gene-expression profiles, or spatial and cellular microenvironmental features [11,14,20,21]. Jacobs et al. examined apheresis T-cell fitness, Deng et al. related the composition and transcriptional state of the manufactured infusion product to clinical and early molecular response, while Pu et al. identified a systemic inflammatory biomarker association [15,22,24]. Plaks et al. showed that CD19-low or CD19-negative tumors accounted for only a subset of evaluable relapses [16]. Chong et al. observed responses to post-CAR T-cell PD-1 blockade in a minority of patients, whereas Jacobson et al. did not establish an incremental benefit from adding PD-L1 blockade to axi-cel [17,23]. Together, these findings did not identify a universal checkpoint-dependent mechanism of resistance.

The available evidence was compatible with multifactorial treatment failure, but heterogeneous sampling and incomplete serial assessment of CD19 expression precluded estimating the relative contributions of antigen escape, T-cell dysfunction, and microenvironmental suppression. Moreover, associations with nonresponse, EFS, or PFS did not necessarily distinguish mechanisms of primary resistance from those of relapse after an initial response. These limitations informed the comparison with published reviews in Section 3.8.

### 3.8. Comparison with Published Reviews According to Disease Scope and Mechanistic Emphasis

Nine published reviews were compared with the clinical and translational findings extracted from the 14 original studies summarized in Table 1 and Table 2. The comparison considered disease focus, classification of treatment failure, mechanisms of resistance, integration of molecular and clinical evidence, and translational contributions (Table 3). The reviews differed substantially in scope: Xu et al. and Aparicio-Pérez et al. concentrated predominantly or exclusively on B-cell acute lymphoblastic leukemia (B-ALL), Byrne et al. specifically addressed LBCL, and Xie et al. and Huang et al. considered multiple B-cell malignancies, although with differing emphasis on leukemia-associated mechanisms [9,25,26,27,28]. Rejeski et al., Bock et al., Zhang et al., and Wang et al. focused on LBCL or DLBCL, with Rejeski et al. also addressing multiple myeloma [29,30,31,32]. These differences were relevant to the interpretation and transferability of their conclusions to LBCL.

#### 3.8.1. LBCL-Focused and Multilevel Reviews

Byrne et al. provided the most directly disease-aligned comparison by focusing on CAR T-cell failure in LBCL [9]. Their objective was to connect potential biological mechanisms with clinical patterns of primary resistance, early relapse, and late relapse. They organized failure into tumor-intrinsic, host-related, and CAR T-cell-related factors and discussed tumor burden, inhibitory signaling, product quality, expansion, persistence, and exhaustion, in addition to CD19 loss. Their distinctive contribution was the integration of these biological categories with a time-based approach to salvage management. The findings summarized in Table 1 and Table 2 are consistent with this multilevel framework and add more detailed correlative evidence linking gene-expression profiles, immune-cell spatial relationships, and microenvironmental states to treatment outcomes.

Rejeski et al. proposed a multidimensional framework of CAR T-cell resistance encompassing host-related, tumor-intrinsic, microenvironmental, systemic inflammatory, and CAR T-cell-related determinants in LBCL and multiple myeloma [29]. Their review emphasized that treatment failure cannot be explained solely by antigen loss, but reflects interactions among tumor burden and genomic complexity, systemic inflammation, an immunosuppressive microenvironment, and inadequate CAR T-cell expansion or persistence. The authors also highlighted potentially modifiable host factors, including physical fitness, hematopoietic reserve, body composition, and the intestinal microbiome. This framework supports our LBCL-focused findings while our review provides greater study-level resolution by linking clinical outcomes to biological characteristics measured in distinct compartments.

Bock et al. reviewed landmark trials and large real-world cohorts evaluating axi-cel, tisa-cel, and liso-cel in LBCL [30]. Across these datasets, unfavorable efficacy and increased toxicity were consistently associated with high tumor burden, elevated systemic inflammatory biomarkers, impaired performance status, and more differentiated T cells in leukapheresis products. Outcomes reported in real-world populations were generally comparable to those of pivotal trials. These clinically measurable correlates complement the present synthesis, which links outcomes to biological findings obtained from distinct compartments.

Zhang et al. presented a DLBCL-focused framework in which resistance to CAR T-cell therapy results from interactions among tumor-mediated mechanisms, CAR T-cell structural and functional limitations, and an immunosuppressive tumor microenvironment [31]. The review emphasized CD19 alterations, clonal evolution, apoptosis and immunological-synapse defects, T-cell exhaustion and metabolic dysfunction, and suppressive immune and stromal components, while discussing multi-targeting, CAR engineering, and combination therapies as potential strategies to overcome resistance. Whereas that review provides a mechanism-oriented taxonomy, the present synthesis anchors selected mechanisms to study-level clinical outcomes in defined LBCL cohorts.

Wang et al. provided a clinically anchored synthesis of CD19-directed CAR T-cell therapy in LBCL, integrating pivotal trials, long-term follow-up, patient selection, and real-world evidence with mechanisms of resistance and toxicity [32]. Treatment failure was characterized as a systems-level process involving antigen modulation, tumor-intrinsic evolution, impaired CAR T-cell fitness, suppressive myeloid and stromal networks, systemic inflammation, metabolic stress, and incomplete immune recovery. The present review shares this systems-level perspective but focuses on original studies with explicit links between outcomes and biomarkers.

#### 3.8.2. Antigen-Escape-Focused and Broader B-Cell Malignancy Reviews

Xu et al. aimed to explain both CD19-positive and CD19-negative relapse and to relate these phenotypes to preventive and therapeutic strategies [25]. Their framework distinguished inadequate CAR T-cell persistence or function from tumor escape through antigen loss or lineage alteration. Accordingly, the review addressed starting T-cell phenotype, expansion, potency, exhaustion, and inhibitory receptors alongside *CD19* mutations, aberrant splicing, immune selection, lineage switching, and trogocytosis. Its principal contribution was an early integration of functional CAR T-cell failure and antigen escape within a mechanism-oriented therapeutic framework. The present synthesis shares this distinction but incorporates more recent LBCL evidence concerning apheresis T-cell metabolic fitness, pretreatment tumor characteristics, and spatially organized immune suppression.

Xie et al. reviewed relapse mechanisms across B-cell hematological malignancies and linked them to potential preventive and therapeutic interventions [27]. Their antigen-positive versus antigen-negative classification encompassed impaired T-cell fitness, manufacturing-related factors, limited persistence, immunogenicity, inhibitory checkpoints, and a suppressive tumor microenvironment, as well as antigen downregulation, masking, mutation, alternative splicing, and lineage switching. The review therefore already recognized substantial interactions between tumor escape and inadequate cellular immunity. Compared with this broad synthesis, the present review concentrates on LBCL and connects selected components of that framework to study-level clinical endpoints and measurable biomarkers, including apheresis T-cell mitochondrial potential, circulating TGF-β1, and tumor gene-expression signatures.

Aparicio-Pérez et al. addressed a narrower question: the mechanisms, frequency, clinical consequences, and prevention of CD19-negative relapse in B-ALL [26]. Their contribution was to integrate leukemia-intrinsic escape biology with clinical-trial and real-world observations, including the selection of pre-existing antigen-negative clones, acquired CD19 alterations, epitope masking, low antigen density, trogocytosis, and lineage switching. The review also incorporated transcriptomic and single-cell evidence and related these mechanisms to alternative-target and multitarget CAR T-cell strategies. Its emphasis differs from that of the present synthesis because it examines a defined antigen-negative leukemia phenotype rather than the broader spectrum of primary resistance and relapse in LBCL. It consequently provides complementary evidence rather than a competing explanation for the microenvironmental and T-cell-associated findings in Table 1 and Table 2.

Huang et al. further developed the analysis of CD19-negative relapse by integrating contemporary molecular evidence with a diagnostic and management framework [28]. The review considered pre-existing antigen-negative or progenitor-like subclones, acquired CD19 alterations, transcriptional and epigenetic regulation, post-transcriptional silencing, altered protein processing, antigen internalization or transfer, and lineage plasticity. Its principal contribution was to connect these mechanisms with serial sampling, detailed immunophenotypic classification, molecular confirmation, and mechanism-directed salvage strategies. Although lymphoma evidence was meaningfully included, much of the detailed clinical guidance concerned B-ALL, particularly lineage-switch-associated disease. The lymphoma evidence was less comprehensive, limiting direct extrapolation of leukemia-specific lineage-switch findings to LBCL.

Across the nine reviews, the comparison identified continuity rather than a simple transition from an antigen-escape model to an immune-biological model. T-cell dysfunction and inadequate persistence were already established components of the earlier frameworks, and microenvironmental suppression was explicitly incorporated by Byrne et al., Xie et al., Rejeski et al., Bock et al., Zhang et al., and Wang et al. [9,27,29,30,31,32]. The present structured narrative review linked these concepts to the selected LBCL clinical and translational evidence: tumor immune contexture in Scholler et al. [11]; outcome-associated gene-expression signatures in Tian et al. [14]; spatial inhibitory interactions in Hirayama et al. [20]; microenvironmental archetypes in Li et al. [21]; apheresis T-cell fitness in Jacobs et al. [22]; infusion-product composition in Deng et al. [15]; CD19 status at relapse in Plaks et al. [16]; and systemic cytokine associations in Pu et al. [24]. The checkpoint-inhibition studies by Chong et al. [17] and Jacobson et al. [23] further qualify the therapeutic implications of exhaustion-associated signaling by showing that targeting the PD-1/PD-L1 axis alone is insufficient to universally overcome treatment failure, likely due to compensatory inhibitory networks or irreversible exhaustion.

Overall, Table 3 identifies a shared multilevel understanding of CAR T-cell resistance, with differences in disease context, mechanistic emphasis, and the resolution of available evidence. The 14-study synthesis supports the relevance of tumor-intrinsic susceptibility, local immune and stromal organization, T-cell fitness, and systemic inflammatory or metabolic conditions in LBCL. However, heterogeneous study designs, overlapping populations, and incomplete assessment of antigen status preclude determining the relative frequency or dominance of these mechanisms. The comparison therefore supports a clinically anchored refinement of existing resistance frameworks, rather than replacement of antigen escape or definitive evidence that a single alternative pathway predominates. 

## 4. Discussion

### 4.1. Principal Findings and Contribution

This review integrates clinical outcomes with biological findings from 14 original studies of CD19-directed CAR T-cell therapy in relapsed or refractory LBCL. The evidence supports a multilevel model in which treatment failure reflects interactions among tumor-intrinsic susceptibility, antigen expression, the cellular fitness of the starting and manufactured products, systemic inflammatory or immunosuppressive signals, and the cellular and spatial organization of the tumor microenvironment. No single mechanism was sufficiently prevalent, consistently measured, or independently validated to explain resistance across studies.

**Figure 1:**
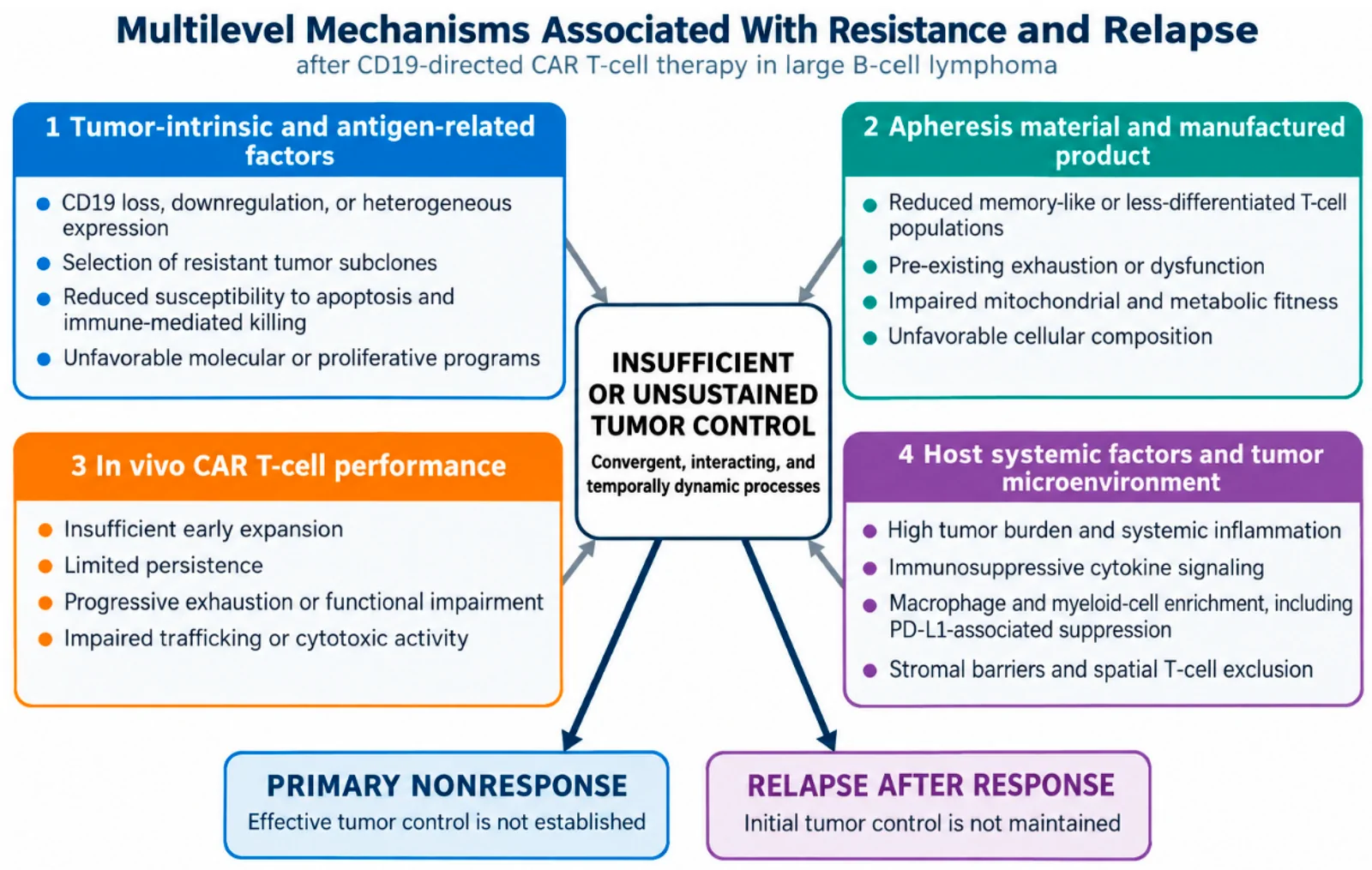
Multilevel mechanisms associated with resistance and relapse after CD19-directed CAR T-cell therapy in large B-cell lymphoma. Treatment failure may arise through interacting factors involving: (1) tumor-intrinsic characteristics and CD19 expression; (2) the composition and functional fitness of the apheresis material and manufactured CAR T-cell product; (3) in vivo CAR T-cell expansion, persistence, trafficking, and dysfunction; and (4) systemic inflammatory signals and the tumor microenvironment, including immunosuppressive myeloid cells, macrophage-associated signaling, stromal barriers, and spatial T-cell exclusion. These mechanisms may prevent the establishment of effective antitumor activity, resulting in primary nonresponse, or undermine initially successful tumor control, contributing to subsequent relapse. The relative contribution of each mechanism remains uncertain because most available evidence is associative, and several studies did not distinguish primary nonresponse from post-response relapse. Blue, teal, orange, and purple distinguish the four mechanistic domains, respectively. Gray arrows indicate their potential convergence on insufficient or unsustained tumor control, while dark blue arrows connect this central state to primary nonresponse or relapse after response. Arrows represent a conceptual framework rather than established causal relationships.

The added value of the present review lies not in proposing a previously unrecognized mechanism, but in connecting established mechanistic categories to study-level clinical observations obtained from distinct biological compartments and time points. Pretreatment tumor transcriptomics and spatial architecture, apheresis-material fitness, infusion-product composition, circulating cytokine trajectories, post-infusion expansion, and relapse-associated antigen changes are considered within one LBCL-specific framework. This organization clarifies what each study can support while avoiding the assumption that correlations derived from different cohorts represent interchangeable or independently replicated mechanisms (Figure 1).

### 4.2. Clinical Context and Heterogeneity of Treatment Failure

Primary nonresponse and relapse after an initial response are biologically related but clinically distinct outcomes. Primary nonresponse indicates that effective antitumor activity was never established and may reflect pre-existing tumor resistance, unfavorable product characteristics, inadequate early expansion, high disease burden, systemic inflammation, or an initially suppressive microenvironment. By contrast, post-response relapse implies that clinically meaningful activity was achieved but not sustained, potentially because of antigen loss or reduced density, selection of resistant subclones, limited CAR T-cell persistence, progressive exhaustion, or re-establishment of immunosuppressive cellular interactions. These processes may coexist, but studies using composite endpoints such as EFS or PFS cannot reliably assign an association to one failure phenotype.

The clinical studies define the magnitude and timing of this unresolved problem. Locke et al. reported that ZUMA-7 established the superiority of second-line axi-cel over standard salvage therapy for early relapsed or refractory LBCL, yet primary progression and early relapse remained frequent [13]. Abramson et al., in TRANSCEND NHL 001, similarly demonstrated durable liso-cel activity in a subset of heavily pretreated patients, while the persistence of early events showed that product standardization and favorable toxicity do not eliminate biological resistance [19]. The findings of Ram et al. in older patients and Ghilardi et al. regarding alternative lymphodepletion further indicate that chronological age or a single preparative regimen does not adequately explain failure [12,18]. These clinical-context studies therefore establish where resistance occurs and identify modifiers of outcome, but they should not be interpreted as direct demonstrations of specific molecular mechanisms.

### 4.3. Tumor-Intrinsic and Microenvironmental Determinants

Four complementary studies place the pretreatment lymphoma ecosystem at the center of response heterogeneity. Scholler et al. associated an immune-inflamed contexture and greater T-cell density with durable benefit, whereas Tian et al. identified gene-expression signatures linked to favorable or unfavorable axi-cel outcomes [11,14]. Hirayama et al. added spatial resolution by showing that the proximity of PD-1-positive T cells to PD-L1-positive macrophages or tumor cells was more informative than immune-cell abundance alone [20]. Li et al. extended this concept by defining lymphoid-enriched, fibroblast/macrophage-enriched, and T-cell-exhausted microenvironmental archetypes with different clinical outcomes [21]. Collectively, these studies suggest that cellular composition, functional state, and spatial organization jointly influence CAR T-cell activity.

This convergence should nevertheless be interpreted cautiously. The studies interrogated different biological layers, used selected biospecimens, and in some instances analyzed overlapping ZUMA-1 or ZUMA-7 populations. Their findings are therefore conceptually consistent but do not constitute independent proof that the TME is universally the dominant cause of failure. Plaks et al. further showed that reduced or absent CD19 contributes to only a subset of relapses, whereas CD19-positive progression may occur when CAR T-cell expansion is insufficient relative to tumor burden [16]. These observations support reassessment of CD19 at relapse while reinforcing that antigen status alone cannot account for most treatment failures in LBCL.

### 4.4. T-Cell Fitness, Systemic Inflammation, and Checkpoint-Directed Interventions

The studies of Jacobs et al. and Deng et al. broaden the tumor-centered model by demonstrating that clinically relevant heterogeneity is also present before and after manufacturing [15,22]. Jacobs et al. associated memory-like CD27-positive CD8-positive cells and greater mitochondrial fitness in the apheresis material with subsequent expansion and response [22]. Deng et al. related memory-associated states in the infusion product to complete response, whereas exhausted CD8-positive and CD4-positive states were enriched in patients with partial response or progressive disease [15]. Together, these findings support a continuum in which the quality of the starting T-cell compartment influences the functional competence of the final product. Both analyses were exploratory and based on selected cohorts, however, and neither established validated clinical thresholds.

Pu et al. added a systemic dimension by associating baseline TGF-β1 and longitudinal inflammatory profiles with toxicity and EFS [24]. These data suggest that systemic immune dysregulation may affect both treatment tolerance and disease control, but they do not establish whether TGF-β1 is a causal mediator, a marker of aggressive disease or tumor burden, or both. The checkpoint-intervention studies provide a parallel caution. Chong et al. reported that pembrolizumab after CAR T-cell progression produced responses and CAR T-cell re-expansion in only a minority of patients, while Jacobson et al. found that adding atezolizumab to axi-cel did not demonstrate a clear incremental benefit over historical experience [17,23]. Thus, exhaustion-associated signaling may remain reversible in selected patients, but blockade of the PD-1/PD-L1 axis alone is unlikely to overcome compensatory inhibitory pathways, fixed dysfunctional states, antigen escape, or unfavorable tumor architecture.

### 4.5. Relationship to Previous Reviews and Translational Implications

Comparison with the nine reviews summarized in Table 3 places the present synthesis within an evolving but continuous conceptual framework. Earlier reviews by Xu et al., Byrne et al., and Xie et al. already distinguished antigen-positive failure related to inadequate CAR T-cell function or persistence from antigen-negative tumor escape [9,25,27]. Subsequent reviews by Rejeski et al. and Bock et al. incorporated tumor burden, host fitness, systemic inflammation, suppressive myeloid and stromal networks, product quality, and metabolic dysfunction, whereas Aparicio-Pérez et al. and Huang et al. provided greater resolution of antigen-negative escape [26,28,29,30]. Zhang et al. provided a detailed DLBCL-centered molecular taxonomy, whereas Wang et al. integrated efficacy, resistance, toxicity, survivorship, biomarkers, and emerging platforms across the treatment pathway [31,32]. The field has therefore not simply replaced an antigen-escape model with an immune-biological model; rather, increasing biological resolution has revealed how several established mechanisms interact.

Within this literature, the distinctive contribution of our review is the structured linkage of mechanistic domains to clinical outcomes reported in selected original LBCL studies. This approach also exposes important gaps. Relapse evaluation should include CD19 reassessment whenever tissue is available, but future biomarker strategies must integrate pretreatment tumor tissue, apheresis material, the manufactured product, post-infusion peripheral blood, and matched relapse tissue. Longitudinal sampling is required to determine whether a biomarker predates therapy, emerges under treatment pressure, or merely accompanies progression. Mechanism-directed interventions should likewise be matched to the relevant biology: alternative or dual targeting for confirmed antigen escape, approaches that preserve memory and metabolic fitness for product-related limitations, and microenvironmental or checkpoint modulation for demonstrable immune suppression.

### 4.6. Limitations and Future Directions

Several limitations constrain the certainty of this synthesis. Because this was a structured narrative rather than a systematic review, the 14 original studies were selected for their relevance to the proposed clinical and biological framework and do not represent an exhaustive evidence base. This relevance-based approach introduces selection bias. The included publications also differed markedly in design, treatment setting, CAR T-cell product, endpoint definition, follow-up, and analytic purpose. Some provided clinical context or tested interventions intended to overcome resistance rather than directly investigating resistance mechanisms.

The mechanistic evidence was predominantly correlative, and many analyses used small or selected cohorts defined by specimen availability or response status. Overlapping trial populations reduced the independence of apparently convergent findings, and incomplete serial assessment of CD19 prevented estimation of the relative frequency of antigen-dependent and antigen-independent failure. Differences among CR, ORR, EFS, PFS, DOR, progression, and relapse further limited direct comparison and prevented quantitative pooling. Larger prospective studies should distinguish primary nonresponse from post-response relapse, use standardized endpoints, and incorporate longitudinal, compartment-specific multi-omic and spatial sampling followed by independent validation. Such designs will be necessary to move from biologically plausible associations toward clinically actionable predictors and mechanism-matched interventions.

## 5. Conclusions

Resistance and relapse after CD19-directed CAR T-cell therapy in LBCL are best understood as context-dependent, multilevel processes rather than consequences of a single biomarker or pathway. The available evidence links treatment failure to tumor-intrinsic susceptibility and antigen expression, the memory and metabolic fitness of the starting and manufactured T-cell products, CAR T-cell expansion and persistence, systemic inflammatory or immunosuppressive signals, disease kinetics, and the cellular and spatial organization of the tumor microenvironment. Preserved CD19 in many treatment failures and the limited activity of checkpoint inhibition in unselected patients indicate that neither antigen escape nor PD-1/PD-L1-mediated dysfunction provides a universal explanation. Primary nonresponse and post-response relapse should therefore be analyzed separately whenever study design and clinical data permit.

The principal contribution of this review is to connect these established mechanistic domains with study-level clinical observations from defined LBCL cohorts and biological compartments. However, heterogeneous designs, selected or overlapping populations, incomplete evaluation of CD19 at relapse, and predominantly correlative analyses prevent ranking the mechanisms by prevalence or causal importance. Future prospective studies should use standardized failure phenotypes and longitudinally integrate pretreatment tumor tissue, apheresis material, the manufactured product, post-infusion peripheral blood, and matched relapse specimens. Independent validation of such multidimensional biomarkers will be essential for patient stratification and mechanism-matched interventions, while avoiding premature exclusion of patients from potentially effective CAR T-cell therapy.

## Figures and Tables

**Table 1:** Study design, patient population, treatment, and biological focus of the 14 original studies included in the structured narrative synthesis.

Study	Journal	Study Type (1, 2) *	Study Design and Evidence Type	Population and LBCL Subtype(s)	CAR T-Cell Product or Intervention	Biological Domain or Study Objective	Relevant Analytical Cohort, *n*	Age, Median or Mean, Years
Deng et al. [15]	Nature Medicine	1	Translational single-cell study with a 24-patient discovery cohort and a 16-patient validation cohort	r/r LBCL: discovery cohort included 16 DLBCL, 6 transformed FL, and 2 PMBCL	Standard-of-care axi-cel	Infusion-product composition and T-cell state assessed by scRNA-seq and CapID+; early molecular response assessed by circulating tumor DNA	24 (response analyses); 16 additional patients (ICANS validation only)	Mean 57 (range, 24–76) across all 40 patients
** Plaks et al. [16]	Blood	1	Translational/correlative analysis of tumor biopsies from the multicenter Phase 1/2 ZUMA-1 trial	Patients with refractory LBCL, including DLBCL, transformed follicular lymphoma, and PMBCL	Axi-cel	Evaluate CD19 protein expression, transcript abundance, alternative splicing, and mutations before treatment and after relapse to characterize antigen-mediated resistance	100 pretreatment biopsies; 20 post-relapse biopsies, including 18 paired pretreatment–relapse samples	NR
Ram et al. [12]	Haematologica	2	Retrospective cohort study (matched control)	DLBCL	Tisa-cel and axi-cel	T-cell subpopulations (CD4/CD8 ratio, naïve, memory, and exhaustion and immune checkpoints, focusing on HLA-DR and Programmed Death 1 (PD-1) on CD4+ and CD8+ T cells) and CAR-T cell expansion	82; 41 older and 41 matched younger controls	Mean: 76.2 (elderly group); Mean: 55.4 (control group)
Chong et al. [17]	Blood	2	Prospective Phase I/II	11 patients with DLBCL and 1 FL patient	Pembrolizumab after CAR-T	PD-1 blockade in reversing T-cell exhaustion and promoting CAR T-cell re-expansion and activation	12	Median: 58
Ghilardi et al. [18]	Annals of Oncology	2	Multicenter retrospective comparative cohort study	Relapsed/refractory LBCL: DLBCL NOS, HGBCL NOS, HGBCL with MYC and BCL2 and/or BCL6 rearrangements, and transformed follicular lymphoma	Tisagenlecleucel after bendamustine versus fludarabine/cyclophosphamide (Flu/Cy) lymphodepletion	Comparative efficacy, survival, lymphocyte depletion and recovery, CAR T-cell-related toxicities, cytopenias, infections, and hospital utilization according to lymphodepletion regimen	132; 90 bendamustine and 42 Flu/Cy	Median: 65 years (IQR, 56–70)
Locke et al. [13]	The New England Journal of Medicine	2	Phase III randomized trial (ZUMA-7)	DLBCL: 69%, including sub-types (histiocyte, Epstein–Barr Virus, cutaneous); HGBCL: 16% and tFL: 13%.	Axi-cel vs. SOC	The trial evaluated whether administering axi-cel immediately as a second-line treatment could avoid salvage chemoimmunotherapy	180 axi-cel; 179 SOC	Median: 59
Scholler et al. [11]	Nature Medicine	1	Translational (ZUMA-1 phase 2 trial)	51 patients with r/r LBCL; DLBCL-specific number not reported	Axi-cel	Tumor immune contexture and TME	51	NR (The overall ZUMA-1 phase 2 cohort had a median age of 58 years)
Abramson et al. [19]	Blood	2	Multicenter, open-label, seamless Phase 1/2 TRANSCEND trial	Relapsed/refractory large B-cell lymphoma (R/R LBCL), including DLBCL, HGBCL, PMBCL, and FL grade 3B	Liso-cel	CD19-directed 4-1BB CAR T cells formulated in a fixed 1:1 ratio of CD4+ and CD8+ components	270 treated; 257 efficacy-evaluable	Median: 63 years (range: 18–86)
Hirayama et al. [20]	HemaSphere	1	A translational/correlative study analyzing gene expression profiling and multiplex immunohistochemistry from pretreatment tumor biopsies to find biological features that correlate with clinical outcomes.	Mainly DLBCL, tHGBCL	CD19 CAR-T cell products: JCAR014 (alone, 12 patients, or with durvalumab, an anti-PD-L1 monoclonal antibody, 8 patients) and JCAR021, a fully human CD19-targeted single-chain variable fragment, 4 patients	Impact of the abundance of PD-L1+ macrophages in the pre-treatment TME on CAR-T therapy efficacy	24	CR: Median age of 65 years; Non-CR: Median age of 59 years
Li et al. [21]	Cancer Cell	1	Translational design combining snMultiome discovery cohort, advanced bioinformatic classification mapping, large-scale external validation sets, and ex vivo functional models to define the TME of LBCL	DLBCL, HGBCL, DHL	Not applicable (observational; validation in axi-cel-treated ZUMA-1 and ZUMA-7 cohorts)	Extract epigenetic and transcriptomic properties at true single-cell resolution: single-nucleus RNA sequencing and single-nucleus ATAC sequencing to analyze 1,886,312 high-quality cells.	19 (primary CAR T analytical cohort); externally validated in ZUMA-1 and ZUMA-7 (*n* = 256, combined treatment arms)	NR
Tian et al. [14]	Frontiers in Oncology	1	Post hoc analysis of the Phase 3 ZUMA-7 trial	r/r LBCL	Axi-cel	Pretreatment tumor gene-expression signatures associated with EFS, PFS, and DOR	134 biomarker-evaluable patients	NR
Jacobs et al. [22]	Blood Immunology & Cellular Therapy	1	Translational/correlative study (from TRANSCEND-NHL-001 clinical trial)	LBCL	Liso-cel	Apheresis T-cell memory composition, mitochondrial fitness, CAR T-cell expansion, and response.	34 selected; 17 responders and 17 nonresponders	NR
Jacobson et al. [23]	Clinical Cancer Research	2	An open-label, single-arm, multicenter, Phase 1/2 trial (ZUMA-6)	DLBCL	Axi-cel + atezolizumab	Test if blocking the PD-1/PD-L1 exhaustion pathway could boost the expansion and long-term effectiveness of the CAR T cells	34 *** treated; 28 (mITT efficacy cohort)	Median: 58
Pu et al. [24]	Chemical Science	1	Single-center longitudinal translational observational cohort with analytical platform development and biomarker discovery	DLBCL	Tisa-cel	The study focused on simultaneously tracking 14 essential cytokines closely tied to CAR T-cell biology and systemic hyperinflammation (including IL-6, IFN-α, IL-10, IL-1β, TNF-α, CRP, and TGF-β).	32	NR

* Study type: (1, mechanistic or biomarker; 2, clinical-context or intervention); ** RNA sequencing was feasible in 22 pretreatment biopsies, six paired pretreatment–relapse biopsies, and three unpaired progression biopsies. *** Jacobson reported 34 treated patients in the safety population, but the principal efficacy analysis included 28 patients from phase I cohort 3 and phase II. Six patients from phase I cohorts 1–2 were excluded from that efficacy population (34 patients received axi-cel plus atezolizumab; 28 constituted the mITT efficacy cohort, and 24/34 completed all four atezolizumab doses). The relevant analytical cohort may differ from the total parent-trial population. Selected biomarker cohorts should not be interpreted as representative efficacy populations. NR, not reported.

**Table 2:** Reported clinical outcomes and principal biological findings related to resistance or relapse in the 14 original studies.

Study	CR, *n*/*N* (%)	ORR, *n*/*N* (%)	PR, *n*/*N* (%)	Relapse or Progression, *n*/*N* (%)	Overall Survival	Progression-Free Survival	Event-Free Survival	Response Durability: DOR or DFS	Biological Finding and Proposed Mechanism	Principal Interpretation
Deng et al. [15]	9/24 (38%)	NR	1/24 (4%)	13/24 (54%, but originally reported as 50% with PD at or before 3 months	NR	NR	NR	NR	Complete responders had approximately threefold higher frequencies of memory-signature CD8+ T cells in the axi-cel infusion product. Exhausted CD8+ and CD4+ T-cell states were enriched in patients with PR/PD, and a CD8+ T-cell exhaustion signature was associated with poor day-7 molecular response.	Cellular and transcriptional heterogeneity of the manufactured axi-cel product may contribute to variation in efficacy. Day-7 circulating tumor DNA response may provide an early indicator of subsequent clinical response.
Plaks et al. [16]	NR	NR	NR	20 evaluable post-relapse cases; percentage not estimable	NR	NR	NR	NR; median time to relapse was 9 months for CD19-negative versus 3 months for CD19-positive relapse	Approximately 30% of evaluable relapses were CD19-low or CD19-negative. Among 18 paired biopsies, 5/18 (28%) showed substantially reduced CD19 protein at relapse. CD19 loss was mainly attributed to selection of tumor cells with low or absent CD19 protein rather than alternative splicing or *CD19* mutations. CD19-positive relapse was associated with insufficient CAR T-cell expansion relative to tumor burden.	Axi-cel failure involves at least two mechanisms: CD19-low/negative antigen escape under therapeutic selection and CD19-positive progression associated with inadequate CAR T-cell activity. Preservation of CD20, CD22, and CD79a at relapse supports multiantigen-targeting strategies.
Ram et al. [12]	Elderly: 19/41 (46%); younger cohort: 24/41 (59%)	Elderly: 26/41 (63%); younger cohort: 32/41 (78%)	Elderly: 7/41 (17%); younger cohort: 8/41 (19%)	Elderly: 13/41 (32%); younger cohort: 9/41 (22%)	12 months: elderly: 69%; younger cohort: 53%	Median 3.6 months (95% CI: 1.6–5.6) in elderly; 12-month PFS: 32% (95% CI NR)	NR	NR	Similar expansion of CAR-T cells (*p* = 0.145) and comparable T-cell fitness (CD4/CD8 ratio, naive/exhaustion markers) between elderly and younger patients.	Outcomes of CAR-T cell therapy: age alone should not preclude treatment. Rapidly growing tumors can outpace the initial expansion wave of the CAR T cells. High LDH levels prior to infusion associated with shorter PFS and shorter OS.
Chong et al. [17]	1/12 (8%)	3/12 (25%)	2/12 (17%)	8/12 (67%)	NR	NR	NR	Rare durable CR (>4 years in one patient)	T cell exhaustion and immunosuppressive TME (PD-L1 expression and PD-1/EOMES/CD39/TOX co-expression). Clusters enriched in responders expressed high activation markers (CD26, CD127, CD69) but low levels of terminal T-cell exhaustion markers (like TOX, EOMES, and CD39).	The use of pembrolizumab after CD19 CAR-T cell therapy is safe and can induce clinical responses in a subset of patients, but the overall tumor expression of PD-L1 did not significantly differ between those who derived clinical benefit and those who did not.
Ghilardi et al. [18]	NR	Bendamustine: 45/90 (50%); Flu/Cy: 18/42 (42.9%)	NR	NR	Median OS: not reached with bendamustine versus 10.23 months with Flu/Cy; *p* = 0.030	Median PFS: 3.26 months (95% CI, 2.58–3.54) with bendamustine versus 3.06 months (95% CI, 2.07–4.44) with Flu/Cy; *p* = 0.210	NR	NR	The lymphodepletion regimen may modify the post-infusion immune and toxicity environment. Flu/Cy produced deeper early lymphocytopenia, whereas bendamustine was associated with lower inflammatory markers, less CRS and ICANS, fewer infections, reduced hematologic toxicity, and lower hospital utilization.	Bendamustine and Flu/Cy were associated with similar ORR and PFS before tisa-cel; bendamustine showed a more favorable toxicity profile. The retrospective, nonrandomized design precludes a definitive conclusion of equivalent efficacy or superior survival.
Locke et al. [13]	Axi-cel: 117/180 (65%)	Axi-cel: 150/180 (83%)	Axi-cel: 33/180 (18%)	82/180 (46%); 82/108 (76%) of EFS events were progression events	axi-cel Group: The median OS was not reached during the initial analysis. SOC Group: The median OS was 35.1 months. At 2 years, the estimated survival rate was 52% compared to 61% in the axi-cel arm.	axi-cel Group: The median PFS was 14.7 months. The 24-month progression-free survival rate was 46%. SOC Group: The median PFS was 3.7 months. The 24-month progression-free survival rate was 27%.	axi-cel Group: The median EFS was 8.3 months. At 24 months, the estimated EFS rate was 41%. SOC Group: The median EFS was 2.0 months. At 24 months, the estimated EFS rate was 16%. Highly significant with a hazard ratio (HR) of 0.40 (*p* < 0.001).	DFS was not used as a formal efficacy metric in the ZUMA-7 protocol. Patients who achieved a CR, the estimated median DOR was 28.4 months. In stark contrast, whereas PR, the median DOR was just 1.6 months	The study mentions that resistance mechanisms may be associated with tumor bulk, inflammation, tumor interferon signaling, myeloid-derived suppressor cells, and CD19 target evasion.	Second-line treatment with axi-cel demonstrated significant superiority in terms of event-free survival and overall response compared to standard-of-care in patients with large B-cell lymphoma.
Scholler et al. [11]	59/101 (58%)	84/101 (83%)	NR	NR	NR	NR	NR	NR	T cell exhaustion in the post-treatment TME, increase in immunosuppressive markers (CTLA-4, CCR4, CCL22), and antigen evasion at the time of relapse.	The pre-treatment TME, characterized by high T cell density and specific immune signature (Immunoscore and Immunosign 21), is a critical determinant of clinical efficacy and survival following axi-cel therapy.
Abramson et al. [19]	136/257 (53%)	187/257 (73%)	51/257 (20%)	NR	Median OS: 27.3 mo (48.5 mo for CR cohort); 2-year OS rate: 50.5%	Median PFS: 6.8 mo; 2-year PFS rate: 40.6%	NR	Median DOR: 23.1 mo (26.1 mo for CR cohort); DFS: NR	Precise 1:1 ratio dosing of CD4+/CD8+ components minimizes variations in product composition to optimize clinical activity and mitigate severe toxicity.	Prolonged cytopenia occurred in 37% of patients on Day 29. Most patients recovered to grade ≤ 2 within approximately 1 month.
Hirayama et al. [20]	9/24 (38%)	NR	NR	NR	NR	Median of 2.6 months (95% CI, 1.4–20.0)	NR	NR	Engagement of the PD-1/PD-L1 axis in the pre-treatment tumor microenvironment through spatial proximity between PD-1+ T cells and PD-L1+ macrophages or tumor cells, in addition to an architecture with intercalated immune infiltrate and dysfunctional T cells.	The success of CD19 CAR-T therapy is influenced by the spatial organization and composition of the tumor microenvironment, where the proximity of PD-L1 inhibitory signal is linked to treatment failure.
Li et al. [21]	NR	NR	NR	NR	NR	NR	ZUMA-7 12-mo EFS with axi-cel: LN 71%, FMAC 41%, TEX 39%. axi-cel versus SOC showed benefit in LN and FMAC	NR	The LBCL TME archetypes are shaped by divergent cell–cell communication: TGFβ drives fibroblasts in FMAC, IL7 maintains T cells in LN, and IFNγ with coinhibitory signals drive macrophage super-activation and T cell exhaustion in TEX.	The LBCL TME can be classified into distinct archetypes (LymphoMAPs) representing diverse cellular ecosystems, which are associated with varied patient outcomes following immunochemotherapy and CD19 CAR T-cell therapy.
Tian et al. [14]	NR	NR	NR	NR	NR	High 6-GES: PFS (HR 0.27); High 17-GES: PFS (HR 7.47).	High 6-GES (favorable): EFS (HR 0.27); High 17-GES (unfavorable): EFS (HR 6.12).	High 6-GES: DOR (HR 0.29); High 17-GES: DOR (HR 7.59). DFS not analyzed	Resistance is associated with immune inflammation signatures, escape mechanisms, apoptosis inhibition, and a metabolically restrictive environment.	The study identified two gene expression signatures (6-GES and 17-GES) that can predict the success or failure of axi-cel treatment in patients with large B-cell lymphoma.
Jacobs et al. [22]	Not estimable from the selected cohort *	Not estimable from the selected cohort *	Not applicable *	Not estimable from the selected cohort *	NR	NR	NR	NR	Nonresponse was associated with lower mitochondrial membrane potential and a more differentiated (lower CD27+) apheresis T-cell compartment.	Higher mitochondrial membrane potential in apheresis-derived T cells and superior in vivo CAR T-cell expansion dynamics.
Jacobson et al. [23]	15/28 (54%)	21/28 (75%)	6/28 (21%)	4/28 (14%)	Median OS (Final Analysis): 32.2 months	Median 9.0 months; 48-month PFS 31.5%	NR	Median DOR was 41.4 months	Interactions between PD-1 and PD-L2 may have bypassed the atezolizumab blockade, or different inhibitory pathways (e.g., LAG-3 or TIM-3) might be the true drivers of CAR T-cell exhaustion in these patients.	Adding atezolizumab to axi-cel showed no clear improvement over historical axi-cel outcomes; the contribution of alternative resistance pathways remains uncertain.
Pu et al. [24]	NR	NR	NR	NR	NR	NR	NR	NR	Elevated baseline TGF-β1 may reflect a pre-existing immunosuppressive milieu that disrupts immunoregulatory networks, leading to compensatory hyperactivation and exaggerated inflammatory response (severe CRS) following CAR-T cell infusion.	The study developed a high-sensitivity platform for cytokine profiling and identified baseline TGF-β1 as a promising biomarker to predict severe toxicity and inferior survival outcomes.

* Cohort selected by response status: 17 responders and 17 nonresponders. Clinical outcomes were extracted from heterogeneous populations, treatment settings, follow-up periods, and analytical cohorts and should not be compared quantitatively across rows. Outcomes from selected translational cohorts are distinguished from those of parent trials. NR, not reported; DOR, duration of response; DFS, disease-free survival.

**Table 3:** Scope, conceptual framework, and distinctive contributions of previous reviews compared with the present structured narrative review.

Review	Disease Scope	Main Objective	Resistance Framework	Evidence Emphasized	Distinctive Contribution
Byrne et al. [9]	LBCL, predominantly DLBCL and related aggressive B-cell lymphomas	Relate biological mechanisms of treatment failure to primary resistance, early relapse, late relapse, and salvage management	Tumor-intrinsic, host-related, and CAR T-cell-related determinants	CD19 loss, tumor burden, immunoediting, inhibitory signaling, product quality, expansion, persistence, and exhaustion	Combined an LBCL-specific biological framework with the clinical timing of failure and potential salvage strategies
Xu et al. [25]	Predominantly B-ALL in children and adults; lymphoma addressed secondarily	Explain CD19-positive and CD19-negative relapse and discuss prevention and treatment strategies	CAR T-cell functional failure or limited persistence versus tumor escape through CD19 loss or lineage alteration	CAR T-cell persistence, starting T-cell phenotype, exhaustion, inhibitory receptors, CD19 alterations, alternative splicing, trogocytosis, and lineage switch	Early integration of CAR T-cell functional failure and antigen escape with mechanism-directed preventive and therapeutic strategies
Xie et al. [27]	Multiple B-cell hematological malignancies, including B-ALL and B-cell lymphomas	Summarize relapse mechanisms and mechanism-directed preventive or therapeutic approaches	Antigen-positive relapse associated with inadequate CAR T-cell function versus antigen-negative relapse associated with tumor escape	Starting T-cell fitness, manufacturing, persistence, inhibitory checkpoints, suppressive TME, antigen downregulation, mutations, alternative splicing, masking, and lineage switching	Broad synthesis linking antigen-positive and antigen-negative relapse mechanisms with potential interventions across B-cell malignancies
Aparicio-Pérez et al. [26]	B-ALL in children and adults	Examine the mechanisms, incidence, outcomes, and prevention of CD19-negative relapse	Leukemia-intrinsic antigen escape despite persisting CAR T-cell immune pressure	Pre-existing CD19-negative clones, acquired CD19 alterations, low antigen density, epitope masking, trogocytosis, alternative splicing, and lineage switch	Detailed integration of CD19-negative escape biology, clinical consequences, and alternative- or multitarget CAR T-cell strategies in B-ALL
Rejeski et al. [29]	r/r LBCL and multiple myeloma treated with CD19- or BCMA-directed CAR T-cell therapy	Review clinical outcomes, mechanisms of resistance, strategies to prevent or overcome resistance, and management after relapse	Host-related, tumor-intrinsic, micro- and macroenvironmental, and CAR T-cell-related determinants	Tumor burden and genomic complexity; antigen loss or reduced density; systemic inflammation; suppressive myeloid and regulatory T-cell populations; CAR T-cell expansion, persistence, exhaustion, and immunogenicity; microbiome, hematopoietic reserve, body composition, and physical fitness	Broad multidimensional framework connecting host fitness, systemic inflammation, tumor biology, TME, and CAR T-cell properties with preventive strategies and treatment options after CAR T-cell failure
Bock et al. [30]	LBCL treated with the three approved CD19-directed products: axi-cel, tisa-cel, and liso-cel	Synthesize efficacy, survival, and toxicity outcomes from definitive trials and large real-world cohorts and identify factors associated with these outcomes	Interacting patient-, tumor-, treatment-, and CAR T-cell product-related determinants of efficacy and toxicity	Performance status, age and comorbidities, tumor burden, systemic inflammatory biomarkers, TME, bridging and lymphodepletion therapies, leukapheresis-product composition, CAR T-cell phenotype, expansion, persistence, and product selection	Integrates pivotal-trial and real-world evidence across all three approved products, emphasizing clinically measurable predictors and the complementary value and limitations of clinical-trial and registry data
Huang et al. [28]	CD19-directed CAR T-cell-treated B-cell malignancies; evidence predominantly derived from B-ALL	Integrate contemporary mechanisms of CD19-negative relapse with diagnostic assessment, prevention, and salvage management	Pre-existing or acquired antigen loss, transcriptional or epigenetic regulation, and lineage plasticity	Genomic, epigenomic, transcriptomic, single-cell, clonal-tracing, immunophenotypic, and clinical evidence	Contemporary diagnostic and management framework linking serial antigen assessment and molecular characterization to mechanism-directed treatment
Wang et al. [32]	LBCL, including DLBCL and related aggressive B-cell lymphomas	Integrate clinical trials, long-term follow-up, patient selection, real-world evidence, resistance, toxicity, biomarkers, and precision strategies	Systems-level interaction among antigen modulation, tumor-intrinsic evolution, CAR T-cell dysfunction, suppressive TME, systemic inflammation, metabolic stress, and incomplete immune recovery	Real-world outcomes; CD19 modulation; CAR T-cell fitness and exhaustion; myeloid and stromal suppression; inflammatory and metabolic biomarkers; ctDNA, PET/CT, multi-omics, and emerging CAR platforms	Broad clinically anchored framework linking efficacy, resistance, toxicity, survivorship, and biomarker-guided precision strategies across the CAR T-cell treatment pathway
Zhang et al. [31]	Predominantly DLBCL, with supporting evidence from other hematologic malignancies and experimental models	Provide a comprehensive synthesis of CAR T-cell resistance mechanisms in DLBCL and discuss strategies for prediction, prevention, and therapeutic reversal	Tumor-mediated resistance—including antigen escape, tumor heterogeneity/clonal evolution, and intrinsic resistance—combined with CAR T-cell structural or functional impairment and an immunosuppressive TME	CD19 genetic, epigenetic, splicing, trafficking, and expression alterations; inhibitory checkpoints; apoptosis resistance; defective immunological synapse; CAR design and signaling; exhaustion and metabolic dysfunction; suppressive immune cells, cytokines, metabolic and physical barriers; multi-targeting, CAR engineering, and combination therapies	Detailed DLBCL-focused molecular taxonomy connecting tumor evolution, CAR T-cell dysfunction, and TME-mediated suppression with emerging precision and next-generation therapeutic strategies
**Present review**	r/r LBCL, predominantly DLBCL and related LBCL subtypes	Integrate selected clinical outcomes with biological findings from distinct compartments and compare the resulting synthesis with previous resistance frameworks	Multilevel interaction among tumor-intrinsic susceptibility, antigen expression, T-cell fitness, CAR T-cell functional state, systemic immune signals, and the TME	Pretreatment tumor transcriptomics, spatial TME architecture, apheresis and manufactured-product characteristics, CAR T-cell expansion and exhaustion, circulating cytokines, and mechanism-directed interventions	Contemporary LBCL-focused synthesis connecting clinical observations with compartment-specific biological evidence and identifying the need for longitudinal, integrated biomarker studies

**Abbreviations:** B-ALL, B-cell acute lymphoblastic leukemia; CAR, chimeric antigen receptor; DLBCL, diffuse large B-cell lymphoma; LBCL, large B-cell lymphoma; r/r, relapsed or refractory; TME, tumor microenvironment.

## Data Availability

No new data were created or analyzed in this study. All information discussed in this review was obtained from previously published and publicly available literature. The data supporting the findings of this study are available in the cited references.

## Supplementary Materials

The following supporting information can be downloaded at: https://www.mdpi.com/article/10.3390/cancers18203370/s1. Table S1: Database Search Strategies.

## Abbreviations

DLBCL	Diffuse large B-cell lymphoma
LBCL	large B-cell lymphoma
r/r	relapsed or refractory
CAR	chimeric antigen receptor
axi-cel	axicabtagene ciloleucel
tisa-cel	tisagenlecleucel
liso-cel	lisocabtagene maraleucel
ORR	overall response rate
CR	complete response
PR	partial response
OS	overall survival
PFS	progression-free survival
EFS	event-free survival
DOR	duration of response
SOC	standard of care
GES	gene expression signatures
tHGBCL	Transformed High-Grade B-Cell Lymphoma
FL	Follicular lymphoma
IQR	Interquartile Range
NOS	Not Otherwise Specified
HGBCL	High-Grade B-Cell Lymphoma
TME	Tumor Microenvironment
DHL	Double-Hit Lymphoma
Flu/Cy	Fludarabine + Cyclophosphamide
LN	lymphoid-enriched
FMAC	fibroblast/macrophage-enriched
TEX	T-cell-exhausted
DFS	Disease-Free Survival
PD	Progressive Disease
CI	Confidence Interval
CRS	Cytokine Release Syndrome
ICANS	Immune Effector Cell-Associated Neurotoxicity Syndrome
B-ALL	B-cell Acute Lymphoblastic Leukemia

## References

[1] Sadelain M. (2015). CAR Therapy: The CD19 Paradigm. J. Clin. Investig..

[2] Daniyan A.F.O., Brentjens R.J. (2016). At the Bench: Chimeric Antigen Receptor (CAR) T Cell Therapy for the Treatment of B Cell Malignancies. J. Leukoc. Biol..

[3] Mitra A., Barua A., Huang L., Ganguly S., Feng Q., He B. (2023). From Bench to Bedside: The History and Progress of CAR T Cell Therapy. Front. Immunol..

[4] Neelapu S.S., Locke F.L., Bartlett N.L., Lekakis L.J., Miklos D.B., Jacobson C.A., Braunschweig I., Oluwole O.O., Siddiqi T., Lin Y. (2017). Axicabtagene Ciloleucel CAR T-Cell Therapy in Refractory Large B-Cell Lymphoma. N. Engl. J. Med..

[5] Schuster S.J., Bishop M.R., Tam C.S., Waller E.K., Borchmann P., McGuirk J.P., Jäger U., Jaglowski S., Andreadis C., Westin J.R. (2019). Tisagenlecleucel in Adult Relapsed or Refractory Diffuse Large B-Cell Lymphoma. N. Engl. J. Med..

[6] Abramson J.S., Palomba M.L., Gordon L.I., Lunning M.A., Wang M., Arnason J., Mehta A., Purev E., Maloney D.G., Andreadis C. (2020). Lisocabtagene Maraleucel for Patients with Relapsed or Refractory Large B-Cell Lymphomas (TRANSCEND NHL 001): A Multicentre Seamless Design Study. Lancet.

[7] Wang Y., Lv L., Song Y., Wei X., Zhou H., Liu Q., Xu K., Yan D., Zhang C., Liu S. (2025). Inaticabtagene Autoleucel in Adult Relapsed or Refractory B-Cell Acute Lymphoblastic Leukemia. Blood Adv..

[8] Labanieh L., Mackall C.L. (2023). CAR Immune Cells: Design Principles, Resistance and the next Generation. Nature.

[9] Byrne M., Oluwole O.O., Savani B., Majhail N.S., Hill B.T., Locke F.L. (2019). Understanding and Managing Large B Cell Lymphoma Relapses after Chimeric Antigen Receptor T Cell Therapy. Biol. Blood Marrow Transpl..

[10] Fraietta J.A., Lacey S.F., Orlando E.J., Pruteanu-Malinici I., Gohil M., Lundh S., Boesteanu A.C., Wang Y., O’Connor R.S., Hwang W.-T. (2018). Determinants of Response and Resistance to CD19 Chimeric Antigen Receptor (CAR) T Cell Therapy of Chronic Lymphocytic Leukemia. Nat. Med..

[11] Scholler N., Perbost R., Locke F.L., Jain M.D., Turcan S., Danan C., Chang E.C., Neelapu S.S., Miklos D.B., Jacobson C.A. (2022). Tumor Immune Contexture Is a Determinant of Anti-CD19 CAR T Cell Efficacy in Large B Cell Lymphoma. Nat. Med..

[12] Ram R., Grisariu S., Shargian-Alon L., Amit O., Bar-On Y., Stepensky P., Yeshurun M., Avni B., Hagin D., Perry C. (2022). Toxicity and Efficacy of Chimeric Antigen Receptor T-Cell Therapy in Patients with Diffuse Large B-Cell Lymphoma above the Age of 70 Years Compared to Younger Patients—A Matched Control Multicenter Cohort Study. Haematologica.

[13] Locke F.L., Miklos D.B., Jacobson C.A., Perales M.-A., Kersten M.-J., Oluwole O.O., Ghobadi A., Rapoport A.P., McGuirk J., Pagel J.M. (2022). Axicabtagene Ciloleucel as Second-Line Therapy for Large B-Cell Lymphoma. N. Engl. J. Med..

[14] Tian Y., Budka J., Locke F.L., Westin J.R., To C., Tiwari G., Mao D., Bedognetti D., Shen R.R., Andrade J. (2025). Tumor Gene Expression Signatures Associated with Outcome in Large B−cell Lymphoma Treated with CD19-Directed CAR T−cell Therapy (Axicabtagene Ciloleucel). Front. Oncol..

[15] Deng Q., Han G., Puebla-Osorio N., Ma M.C.J., Strati P., Chasen B., Dai E., Dang M., Jain N., Yang H. (2020). Characteristics of Anti-CD19 CAR T Cell Infusion Products Associated with Efficacy and Toxicity in Patients with Large B Cell Lymphomas. Nat. Med..

[16] Plaks V., Rossi J.M., Chou J., Wang L., Poddar S., Han G., Wang Z., Kuang S.-Q., Chu F., Davis R.E. (2021). CD19 Target Evasion as a Mechanism of Relapse in Large B-Cell Lymphoma Treated with Axicabtagene Ciloleucel. Blood.

[17] Chong E.A., Alanio C., Svoboda J., Nasta S.D., Landsburg D.J., Lacey S.F., Ruella M., Bhattacharyya S., Wherry E.J., Schuster S.J. (2022). Pembrolizumab for B-Cell Lymphomas Relapsing after or Refractory to CD19-Directed CAR T-Cell Therapy. Blood.

[18] Ghilardi G., Chong E.A., Svoboda J., Wohlfarth P., Nasta S.D., Williamson S., Landsburg J.D., Gerson J.N., Barta S.K., Pajarillo R. (2022). Bendamustine Is Safe and Effective for Lymphodepletion before Tisagenlecleucel in Patients with Refractory or Relapsed Large B-Cell Lymphomas. Ann. Oncol..

[19] Abramson J.S., Palomba M.L., Gordon L.I., Lunning M., Wang M., Arnason J., Purev E., Maloney D.G., Andreadis C., Sehgal A. (2024). Two-Year Follow-up of Lisocabtagene Maraleucel in Relapsed or Refractory Large B-Cell Lymphoma in TRANSCEND NHL 001. Blood.

[20] Hirayama A.V., Wright J.H., Smythe K.S., Fiorenza S., Shaw A.N., Gauthier J., Maloney D.G., Naresh K.N., Yeung C.C.S., Turtle C.J. (2024). PD-L1+ Macrophage and Tumor Cell Abundance and Proximity to T Cells in the Pretreatment Large B-Cell Lymphoma Microenvironment Impact CD19 CAR-T Cell Immunotherapy Efficacy. HemaSphere.

[21] Li X., Singhal K., Deng Q., Chihara D., Russler-Germain D., Harkins R.A., Henderson J., Arita K., Kizhakeyil A., Sun R. (2025). Large B Cell Lymphoma Microenvironment Archetype Profiles. Cancer Cell.

[22] Jacobs C.F., Simon-Molas H., Cretenet G., Gutschow M., Collett G., Colonna L., Ogasawara K., Stepan L., Khuu-Duong K., Kater A.P. (2026). Lisocabtagene Maraleucel Outcome in Large B-Cell Lymphoma Associates with Memory Composition and Mitochondrial Fitness of T Cells. Blood Immunol. Cell. Ther..

[23] Jacobson C.A., Westin J.R., Miklos D.B., Herrera A.F., Ritz J., Rodig S., Arihara Y., Cao S., Sun J., Mao D. (2026). Axicabtagene Ciloleucel in Combination with Atezolizumab in Patients with Refractory Diffuse Large B-Cell Lymphoma: The Phase 1/2 ZUMA-6 Trial. Clin. Cancer Res..

[24] Pu K., He W., Feng J., Li H., Chan C.-L., Chan T.S.Y., Tse E., Sun H. (2026). High-Throughput Bead-Based Cytokine Profiling Reveals Dynamic Immune Signatures and Identifies Candidate Exploratory Biomarkers in CAR-T Therapy. Chem. Sci..

[25] Xu X., Sun Q., Liang X., Chen Z., Zhang X., Zhou X., Li M., Tu H., Liu Y., Tu S. (2019). Mechanisms of Relapse After CD19 CAR T-Cell Therapy for Acute Lymphoblastic Leukemia and Its Prevention and Treatment Strategies. Front. Immunol..

[26] Aparicio-Pérez C., Carmona M., Benabdellah K., Herrera C. (2023). Failure of ALL Recognition by CAR T Cells: A Review of CD 19-Negative Relapses after Anti-CD 19 CAR-T Treatment in B-ALL. Front. Immunol..

[27] Xie D., Jin X., Sun R., Zhang M., Wang J., Xiong X., Zhang X., Zhao M. (2022). Relapse Mechanism and Treatment Strategy After Chimeric Antigen Receptor T-Cell Therapy in Treating B-Cell Hematological Malignancies. Technol. Cancer Res. Treat..

[28] Huang J., Huang X., Yu D. (2026). CD19-Negative Relapse after CAR-T Cell Therapy: Mechanisms of Antigen Escape and Lineage Switch. Front. Immunol..

[29] Rejeski K., Jain M.D., Smith E.L. (2023). Mechanisms of Resistance and Treatment of Relapse after CAR T-Cell Therapy for Large B-Cell Lymphoma and Multiple Myeloma. Transplant. Cell. Ther..

[30] Bock T.J., Colonne C.K., Fiorenza S., Turtle C.J. (2025). Outcome Correlates of Approved CD19-Targeted CAR T Cells for Large B Cell Lymphoma. Nat. Rev. Clin. Oncol..

[31] Zhang S., Liu J., Li Z. (2026). Research Progress on Resistance Mechanisms to CAR-T Cell Therapy in Diffuse Large B-Cell Lymphoma. Front. Oncol..

[32] Wang C., Zhu J., Qiao J., Wang H., Liang X. (2026). CD19 CAR-T Cell Therapy in Large B-Cell Lymphoma: Clinical Evidence, Resistance, Toxicity, and Precision Strategies. Front. Immunol..

