# Supplementary material for: Clinical and Biological Correlates of Resistance and Relapse After CD19-Directed CAR T-Cell Therapy in Large B-Cell Lymphoma: A Structured Narrative Review
Source: Cancers (Basel). 2026 Oct 18;18(20):3370. doi: 10.3390/cancers18203370 (PMC13702201; doi:10.3390/cancers18203370)
Supplement: Supplementary file 1 [file cancers-18-03370-s001.zip › cancers-4596093-supplementary.pdf]

Supplementary Table S1 Database Search Strategies

Database-specific strategies used to identify original clinical and translational studies addressing clinical or biological correlates of resistance, refractory disease, or relapse after CD19-directed CAR T-cell therapy in adults with large B-cell lymphoma.

Database	Complete search strategy	Coverage and filters
**PubMed/MEDLINE**	("Lymphoma, Large B-Cell, Diffuse"[Mesh] OR "large B-cell lymphoma"[tiab] OR "large B cell lymphoma"[tiab] OR DLBCL[tiab] OR LBCL[tiab] OR "high-grade B-cell lymphoma"[tiab] OR "high grade B cell lymphoma"[tiab] OR "primary mediastinal B-cell lymphoma"[tiab] OR PMBCL[tiab] OR "transformed follicular lymphoma"[tiab]) AND ("Receptors, Chimeric Antigen"[Mesh] OR "CAR T-cell"[tiab] OR "CAR T-cells"[tiab] OR "CAR-T cell"[tiab] OR "CAR-T cells"[tiab] OR "chimeric antigen receptor T-cell"[tiab] OR "chimeric antigen receptor T cells"[tiab] OR axicabtagene[tiab] OR axi-cel[tiab] OR tisagenlecleucel[tiab] OR tisa-cel[tiab] OR lisocabtagene[tiab] OR liso-cel[tiab]) AND (resistan*[tiab] OR refractor*[tiab] OR relaps*[tiab] OR recurren*[tiab] OR failure[tiab] OR response[tiab] OR outcome*[tiab] OR biomarker*[tiab] OR "antigen escape"[tiab] OR "antigen loss"[tiab] OR exhaustion[tiab] OR persistence[tiab] OR fitness[tiab] OR "tumor microenvironment"[tiab] OR "tumour microenvironment"[tiab])	Until 31 July 2026<br>No language or article-type filters unless otherwise documented
**Embase (Embase.com)**	('large b cell lymphoma'/exp OR 'large b cell lymphoma':ti,ab,kw OR 'diffuse large b cell lymphoma':ti,ab,kw OR dlbcl:ti,ab,kw OR lbcl:ti,ab,kw OR 'high grade b cell lymphoma':ti,ab,kw OR 'primary mediastinal b cell lymphoma':ti,ab,kw OR pmbcl:ti,ab,kw OR 'transformed follicular lymphoma':ti,ab,kw) AND ('chimeric antigen receptor t cell therapy'/exp OR 'car t cell':ti,ab,kw OR 'car t cells':ti,ab,kw OR 'car-t cell':ti,ab,kw OR 'car-t cells':ti,ab,kw OR 'chimeric antigen receptor t cell':ti,ab,kw OR axicabtagene:ti,ab,kw OR 'axi-cel':ti,ab,kw OR tisagenlecleucel:ti,ab,kw OR 'tisa-cel':ti,ab,kw OR lisocabtagene:ti,ab,kw OR 'liso-cel':ti,ab,kw) AND (resistan*:ti,ab,kw OR refractor*:ti,ab,kw OR relaps*:ti,ab,kw OR recurren*:ti,ab,kw OR failure:ti,ab,kw OR response:ti,ab,kw OR outcome*:ti,ab,kw OR biomarker*:ti,ab,kw OR 'antigen escape':ti,ab,kw OR 'antigen loss':ti,ab,kw OR exhaustion:ti,ab,kw OR persistence:ti,ab,kw OR fitness:ti,ab,kw OR 'tumor microenvironment':ti,ab,kw OR 'tumour microenvironment':ti,ab,kw)	Until 31 July 2026<br>No language or article-type filters unless otherwise documented
**Web of Science Core Collection**	TS=(("large B-cell lymphoma" OR "large B cell lymphoma" OR "diffuse large B-cell lymphoma" OR DLBCL OR LBCL OR "high-grade B-cell lymphoma" OR "primary mediastinal B-cell lymphoma" OR PMBCL OR "transformed follicular lymphoma") AND ("CAR T-cell*" OR "CAR-T cell*" OR "chimeric antigen receptor T-cell*" OR axicabtagene OR "axi-cel" OR tisagenlecleucel OR "tisa-cel" OR lisocabtagene OR "liso-cel") AND (resistan* OR refractor* OR relaps* OR recurren* OR failure OR response OR outcome* OR biomarker* OR "antigen escape" OR "antigen loss" OR exhaustion OR persistence OR fitness OR "tumor microenvironment" OR "tumour microenvironment"))	Until 31 July 2026<br>No language or document-type filters unless otherwise documented

Database	Complete search strategy	Coverage and filters
**Scopus**	TITLE-ABS-KEY(("large B-cell lymphoma" OR "large B cell lymphoma" OR "diffuse large B-cell lymphoma" OR DLBCL OR LBCL OR "high-grade B-cell lymphoma" OR "primary mediastinal B-cell lymphoma" OR PMBCL OR "transformed follicular lymphoma") AND ("CAR T-cell*" OR "CAR-T cell*" OR "chimeric antigen receptor T-cell*" OR axicabtagene OR "axi-cel" OR tisagenlecleucel OR "tisa-cel" OR lisocabtagene OR "liso-cel") AND (resistan* OR refractor* OR relaps* OR recurren* OR failure OR response OR outcome* OR biomarker* OR "antigen escape" OR "antigen loss" OR exhaustion OR persistence OR fitness OR "tumor microenvironment" OR "tumour microenvironment"))	Until 31 July 2026<br>No language or document-type filters unless otherwise documented

Note. Replace the bracketed fields with the actual date on which each search was performed or rerun and the corresponding number of records retrieved. The Embase syntax shown is for Embase.com and should be adapted if Ovid Embase was used. Reference-list examination and citation tracking were conducted separately.
